# Radiomics and deep learning in upper tract urothelial carcinoma: advancing preoperative risk stratification and clinical decision-making

**DOI:** 10.3389/fonc.2026.1838755

**Published:** 2026-06-19

**Authors:** Yanwei Zhang, Gang Wu, Fengze Sun, Bin Wang, Yicheng Guo, Jitao Wu

**Affiliations:** 1 Second Clinical Medical College, Binzhou Medical University, Yantai, China; 2 Department of Urology, Yantai Yuhuangding Hospital, Yantai, China

**Keywords:** deep learning, preoperative risk stratification, prognostic prediction, radiomics, upper tract urothelial carcinoma

## Abstract

**Background:**

Upper tract urothelial carcinoma (UTUC) is a relatively rare but aggressive malignancy. Accurate preoperative assessment of tumor grade, invasiveness, and prognosis remains challenging using conventional imaging, cytology, and ureteroscopic biopsy alone. Radiomics and deep learning may provide noninvasive tools for improving risk stratification and clinical decision-making.

**Methods:**

This narrative review summarizes current evidence on radiomics, machine learning, and deep learning in UTUC. Relevant studies were identified from PubMed, Web of Science, and Scopus and synthesized according to clinical applications and methodological considerations.

**Results:**

Radiomics and deep learning models have shown promising performance in pathological grade prediction, differentiation between UTUC and renal cell carcinoma, muscle invasion assessment, and survival or recurrence risk stratification. However, most studies remain retrospective, single-center, and limited by small sample sizes, heterogeneous imaging protocols, inconsistent segmentation methods, insufficient external validation, and limited evidence of clinical utility.

**Conclusion:**

Radiomics and deep learning are promising approaches for noninvasive preoperative risk stratification in UTUC. Future studies should focus on methodological standardization, multicenter external validation, prospective evaluation, model interpretability, and demonstration of incremental clinical benefit before routine clinical implementation.

## 1 Introduction

### 1.1 Clinical challenges of UTUC

Urothelial carcinoma (UC) is the second most common malignancy of the genitourinary tract in developed countries and can be anatomically classified into lower tract (bladder and urethra) and upper tract disease (1–3). Upper tract urothelial carcinoma (UTUC), arising from the renal pelvis or ureter, represents approximately 5–10% of all UC cases, with an annual incidence of 1–2 per 100,000 individuals in Western populations (4–6). Notably, marked geographic variation exists, with UTUC accounting for up to 20–30% of urothelial carcinomas in certain Asian regions (7, 8). In Taiwan, for instance, the incidence is among the highest worldwide, partly attributed to long-term exposure to aristolochic acid (9–12). These regional disparities underscore the complex interplay between environmental factors and genetic susceptibility in UTUC pathogenesis.

Despite its relatively low incidence, UTUC is characterized by aggressive biological behavior. Approximately two-thirds of patients present with muscle-invasive or high-grade disease at diagnosis, which is associated with increased risks of recurrence, metastasis, and cancer-specific mortality (4). Compared with bladder cancer, UTUC often demonstrates more rapid progression and poorer long-term outcomes. Therefore, early identification of high-risk patients is critical to optimize therapeutic strategies and improve survival (13).

The diagnostic workup relies on a multimodal approach integrating cross-sectional imaging, endoscopic evaluation, cytology, and histopathological assessment (14). Contrast-enhanced computed tomography urography (CTU) remains the first-line imaging modality, providing detailed information on tumor location, size, enhancement patterns, and potential local extension (15–17). Magnetic resonance urography may serve as an alternative in selected patients, particularly those with contraindications to iodinated contrast agents (18–21). However, both modalities have limited accuracy in differentiating tumor grade and assessing microscopic muscle invasion (22).

Urinary cytology demonstrates high specificity for high-grade disease, yet its sensitivity for low-grade tumors is suboptimal (23–25). Ureteroscopy (URS) allows direct visualization of the upper urinary tract and targeted biopsy, and is currently considered the most important preoperative tool for tumor grading (26–28). Nevertheless, biopsy specimens are often limited in size and depth, potentially leading to underestimation of tumor stage or grade due to sampling error and tumor heterogeneity.

Definitive pathological staging is ultimately established following radical nephroureterectomy or, less commonly, surgical biopsy. Consequently, precise preoperative characterization of tumor aggressiveness remains challenging, creating uncertainty in treatment selection and risk stratification.

A central question therefore arises: can imaging-based artificial intelligence provide clinically actionable information that meaningfully improves preoperative risk assessment beyond existing diagnostic tools? This question is particularly complex in UTUC, given its relatively low incidence, anatomical complexity, thin muscular layer, and limited availability of large annotated datasets (29). These disease-specific characteristics distinguish UTUC from other urologic malignancies and may substantially influence model development, validation, and generalizability.

### 1.2 Emergence of radiomics and deep learning in urologic oncology

Advances in medical imaging and computational power have accelerated the integration of radiomics and deep learning into oncologic imaging analysis. Radiomics transforms conventional images into high-dimensional quantitative data, capturing tumor heterogeneity beyond visual interpretation (30). Deep learning, particularly convolutional neural networks, enables end-to-end learning and has demonstrated strong performance in lesion detection, segmentation, and risk prediction tasks.

In renal cell carcinoma, radiomics models based on contrast-enhanced CT have shown improved prediction of tumor grade and survival outcomes compared with traditional clinical parameters (31, 32). In prostate cancer, imaging-based artificial intelligence has enhanced detection of clinically significant disease and improved consistency in interpretation of multiparametric MRI. Similarly, in bladder cancer, radiomics and deep learning approaches have demonstrated promising performance in predicting muscle invasion and treatment response (33, 34).

Although UTUC shares certain imaging and biological characteristics with other urologic malignancies, its lower incidence and unique anatomical context pose additional challenges for model development and validation. Nevertheless, the successful application of artificial intelligence in related tumors provides a conceptual and methodological foundation for its use in UTUC. A comprehensive understanding of current advances and limitations is therefore essential to guide future research and clinical translation.

The main aim of this narrative review is to summarize current advancements in the application of radiomics and deep learning in UTUC, evaluate the methodological quality of existing studies, and highlight the translational challenges of these technologies. We also discuss future research directions, specifically how to address current methodological limitations to translate these imaging-based artificial intelligence techniques into clinical practice, aiding preoperative risk stratification and personalized treatment decision-making.

## 2 Methods

To provide a comprehensive overview of current radiomics and deep learning applications in UTUC, we conducted a comprehensive literature search to identify relevant studies. This search was performed across multiple databases and included studies that specifically addressed imaging-based artificial intelligence methods, with a focus on UTUC-related clinical tasks such as pathological grade prediction, tumor staging, muscle invasion assessment, and prognostic stratification. Below, we outline the literature search strategy and study selection process that guided the inclusion of studies in this review. It should be noted that this is a narrative review, rather than a formal systematic review.

### 2.1 Literature search strategy

A comprehensive literature search was conducted in PubMed, Web of Science, and Scopus databases to identify relevant articles published up to March 2026.The following search terms were used: “upper tract urothelial carcinoma,” “radiomics,” “machine learning,” “deep learning,” “artificial intelligence,” “computed tomography,” “magnetic resonance urography,” “tumor grade,” “muscle invasion,” “survival,” and “prognosis.” Articles were included if they focused on radiomics, machine learning, or deep learning applications in UTUC. We excluded conference abstracts, articles not written in English, and studies unrelated to imaging-based AI in UTUC. The final selection was based on the relevance to the clinical tasks of pathological grading, tumor staging, muscle invasion prediction, and prognostic stratification.

### 2.2 Radiomics workflow and methodological considerations

Radiomics is a quantitative imaging approach that converts standard medical images into high-dimensional data through systematic feature extraction and computational modeling (35–37). A typical radiomics workflow consists of several key steps: image acquisition, tumor segmentation, feature extraction, feature selection, model construction, and validation (38–41).

**Figure 1:**
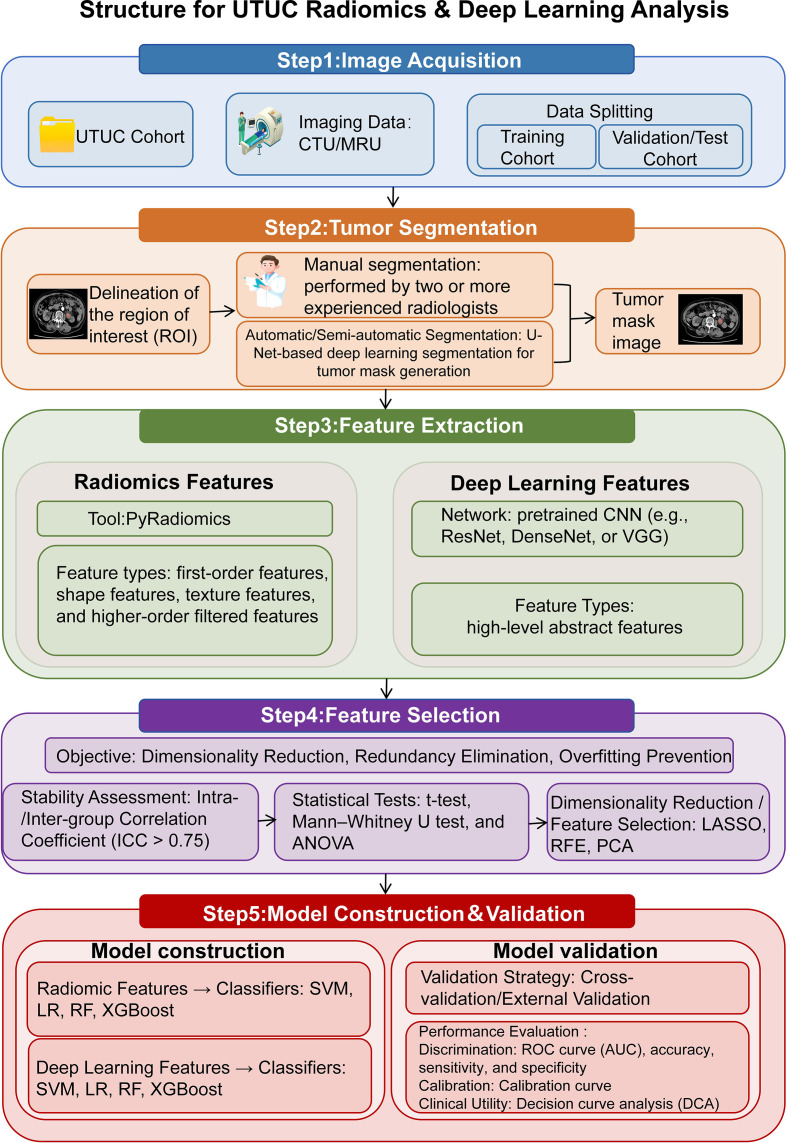
Workflow of UTUC radiomics and deep learning analysis. The overall workflow consisted of five steps: image acquisition, tumor segmentation, feature extraction, feature selection, and model construction and validation. First, imaging data (CTU/MRU) from the UTUC cohort were collected and divided into training and validation/test cohorts. Second, the tumor region of interest (ROI) was segmented manually by experienced radiologists or automatically/semi-automatically using a U-Net-based deep learning approach to generate tumor mask images. Third, radiomics features were extracted using PyRadiomics, including first-order, shape, texture, and higher-order filtered features, while deep learning features were extracted from pretrained convolutional neural networks. Fourth, feature selection was performed to reduce dimensionality, eliminate redundancy, and prevent overfitting through stability assessment, statistical testing, and dimensionality reduction/selection methods. Finally, radiomics features and deep learning features were used independently to construct predictive models, which were then evaluated by cross-validation or external validation. Model performance was assessed in terms of discrimination, calibration, and clinical utility.

The overall workflow for UTUC radiomics and deep learning analysis is summarized in **Figure 1**.

Image acquisition and reconstruction parameters critically influence feature stability. Variability in scanner type, slice thickness, contrast phase, and reconstruction algorithms may introduce substantial heterogeneity, potentially compromising reproducibility across institutions. Standardization of imaging protocols or *post hoc* harmonization techniques is therefore essential for robust model development.

Tumor segmentation represents another crucial step. Regions of interest may be delineated manually, semi-automatically, or automatically. Manual segmentation remains common in UTUC studies but is time-consuming and subject to interobserver variability. Automated segmentation methods have shown promise in other urologic malignancies; however, their application in UTUC remains limited.

Once the region of interest is defined, hundreds to thousands of quantitative features can be extracted, including first-order statistics, shape descriptors, texture features (e.g., gray-level co-occurrence matrix, gray-level run-length matrix), and higher-order filtered features. Given the relatively small sample sizes typical of UTUC research, radiomics studies frequently encounter the classical “high-dimensional, low-sample-size” (p >> n) problem. Without appropriate feature reduction strategies—such as least absolute shrinkage and selection operator (LASSO), principal component analysis, or recursive feature elimination—models are highly susceptible to overfitting.

Model construction generally involves conventional machine learning algorithms, including logistic regression, support vector machines, random forests, or gradient boosting techniques. Internal validation through cross-validation or bootstrapping is commonly performed; however, true external validation using independent multicenter cohorts remains limited in the current UTUC literature. This gap substantially affects the generalizability and clinical credibility of published models.

Beyond technical workflow considerations, methodological quality remains a critical issue in UTUC radiomics research. The Radiomics Quality Score (RQS) has been proposed to systematically evaluate study rigor, including imaging protocol standardization, reproducibility testing, biological validation, and external validation (41–43). However, many published UTUC studies satisfy only a limited proportion of RQS criteria, particularly with respect to prospective design, multicenter validation, and assessment of clinical utility.

Furthermore, risks of data leakage—such as performing feature normalization, feature selection, or segmentation prior to dataset splitting—are inconsistently reported and may artificially inflate model performance. Class imbalance, especially in survival prediction tasks with relatively few outcome events, represents another statistical concern that is often insufficiently addressed. Adherence to reporting frameworks such as TRIPOD-AI and CLAIM is essential to enhance transparency, reproducibility, and clinical credibility in future studies (44).

### 2.3 Deep learning approaches and their distinct characteristics

Deep learning, particularly convolutional neural networks (CNNs), has emerged as an alternative strategy that enables end-to-end learning directly from imaging data (45, 46). Unlike traditional radiomics, which relies on handcrafted feature engineering, deep learning automatically learns hierarchical representations that may capture more complex spatial and contextual information (47).

In oncologic imaging, deep learning has demonstrated strong performance in lesion detection, segmentation, classification, and outcome prediction (48–50). Architectures such as ResNet, DenseNet, and U-Net have been widely applied across urologic cancers. Some UTUC studies have also explored hybrid models integrating CNN-derived features with clinical variables to improve predictive performance.

However, deep learning models typically require large annotated datasets for optimal performance, which presents a significant challenge in UTUC due to its relatively low incidence. Limited sample size increases the risk of model instability and overfitting, particularly when training is performed without independent validation cohorts. Transfer learning and data augmentation strategies may partially mitigate these limitations but cannot fully substitute for robust multicenter datasets.

Another critical consideration is interpretability. Deep learning models are often perceived as “black boxes,” and their decision-making processes may not be readily transparent to clinicians. Visualization tools such as class activation maps or attention mechanisms have been introduced to enhance interpretability, yet their clinical utility remains under investigation. Ensuring transparency and explainability is essential for fostering trust and facilitating integration into surgical decision-making.

## 3 Radiomics and deep learning in clinical practice of UTUC

### 3.1 Pathological grade prediction in clinical practice, accurate

preoperative tumor grading is fundamental for risk stratification and treatment selection in upper tract urothelial carcinoma (UTUC). Distinguishing high-grade from low-grade tumors directly determines whether a patient is eligible for kidney-sparing surgery or should undergo radical nephroureterectomy (RNU) (51–53). According to the European Association of Urology (EAU) guidelines, classification into low-risk and high-risk categories is mandatory before therapeutic decision-making in non-metastatic UTUC (54). High-risk disease generally warrants RNU with bladder cuff excision and lymph node dissection, often followed by adjuvant systemic therapy. In contrast, kidney-sparing surgery is recommended for carefully selected low-risk patients to preserve renal function without compromising oncologic control (54). Despite its clinical importance, reliable preoperative grading remains challenging. Ureteroscopic biopsy, the current standard for preoperative pathological assessment, is limited by sampling error, tumor heterogeneity, and insufficient tissue depth, frequently leading to grade underestimation. In addition, technical limitations and concerns regarding procedure-related tumor dissemination further restrict its universal applicability. Therefore, the development of accurate noninvasive imaging-based grading tools is of substantial clinical significance. Radiomics has been widely applied to CT and MRI for this purpose. Zheng et al. (55) constructed a multiphase CT urography radiomics model integrating features from different enhancement phases, achieving robust discrimination of high-grade UTUC. Nai et al. (56) developed an ADC-based radiomics model and demonstrated that texture-derived features significantly outperformed conventional mean ADC values in grade prediction, highlighting the advantage of high-dimensional feature extraction. Al Mopti et al. (57) further incorporated perirenal fat (PRF) radiomic features together with tumor features and reported an AUC of 0.961 for grade prediction, which was superior to models based solely on tumor or PRF features, suggesting that peritumoral microenvironmental information may provide incremental value. Deep learning approaches have also shown encouraging performance. Alqahtani et al. (58) developed a CT-based deep learning framework that achieved high sensitivity and specificity for grade prediction, further supporting the feasibility of fully automated image-based classification. Although these models report strong discriminatory ability—often with AUC values exceeding 0.80 in internal validation—several methodological concerns remain. Most studies are retrospective and single-center, with relatively limited sample sizes, raising the possibility of overfitting in high-dimensional settings. In addition, many models rely on ureteroscopic biopsy rather than final surgical pathology as the reference standard, which may introduce label misclassification. External validation cohorts are still scarce. Therefore, while existing evidence supports the potential of radiomics and deep learning for noninvasive grade prediction, multicenter validation and prospective assessment are necessary before routine clinical implementation.

### 3.2 Tumor differentiation between UTUC and RCC accurate differentiation

between UTUC and renal cell carcinoma (RCC) is essential because the surgical management strategies differ substantially. UTUC typically requires radical nephroureterectomy with bladder cuff excision (4), whereas RCC is usually treated with partial or radical nephrectomy depending on tumor stage and anatomical considerations (59). However, in cases where tumors involve the renal pelvis or exhibit infiltrative growth, conventional CT findings may overlap, creating diagnostic uncertainty. Radiomics-based models have demonstrated promising discriminatory performance in this context. Marcon et al. (60) extracted standardized radiomic features from preoperative portal venous phase CT images and applied LASSO regression to construct a predictive model. The model achieved an AUC of 0.93 in the training cohort (sensitivity 88.4%, specificity 81%) and 0.87 in the validation cohort (sensitivity 80.6%, specificity 80%), outperforming conventional imaging assessment. Similarly, Zhai et al. (61) focused on differentiating pyelocaliceal UTUC from invasive RCC, a particularly challenging clinical scenario. Their CT-based radiomics model yielded AUCs of 0.95 and 0.90 in the training and testing cohorts, respectively. When combined with clinical factors, the integrated model achieved AUCs of 0.99 and 0.90, significantly surpassing visual interpretation alone. These results indicate that radiomics can capture subtle intratumoral and peritumoral heterogeneity beyond human perception. Nevertheless, most studies remain retrospective and lack prospective head-to-head comparisons with experienced radiologists. Thus, while diagnostic performance metrics are impressive, the real-world incremental value and generalizability require further validation.

### 3.3 Assessment of muscle invasion

Muscle invasion represents a critical determinant of prognosis and treatment intensity in UTUC. However, due to the thin muscular layer of the upper urinary tract and limitations in imaging resolution, accurate preoperative evaluation of muscle-invasive disease remains difficult with conventional CT or MRI. Radiomics-based predictive modeling offers a potential solution. Zhang et al. (62) developed a CT-based radiomics model by extracting 1,781 imaging features and selecting nine key predictors for muscle invasion. The model achieved AUCs of 0.859 in the training cohort and 0.821 in the external validation cohort, indicating stable discriminative performance. When combined with clinical features such as tumor size, the integrated model further improved sensitivity and negative predictive value. These findings suggest that quantitative texture analysis may reveal imaging signatures associated with microscopic invasion. However, variability in segmentation protocols and imaging acquisition parameters may influence reproducibility. Larger multicenter studies are needed to confirm robustness across scanners and populations.

### 3.4 Survival prediction and prognostic stratification

Prognostic prediction in UTUC remains challenging due to tumor heterogeneity and relatively high recurrence rates. Traditional prognostic models primarily rely on postoperative pathological variables, limiting their utility for preoperative counseling and risk-adapted therapeutic planning. Alqahtani et al. (63) constructed a prognostic model integrating CT radiomic features with clinical data. The model achieved a C-index of 0.73 for overall survival prediction and 0.84 for recurrence prediction. Kaplan–Meier analysis demonstrated significant survival differences between predicted risk groups, supporting its clinical stratification capability. Sun et al. (64) developed a multimodal deep learning model (MICC) combining multiphase enhanced CT images with clinical information. The model achieved AUCs of 0.918 and 0.895 in the training and testing cohorts, respectively, outperforming single-modality models. Time-dependent AUC analysis at 12 and 60 months further confirmed its stability in both short- and long-term prognostic prediction. Peng et al. (65) proposed a deep learning framework (PGCA-Net) integrating pathological image features for survival stratification, reporting C-index values ranging from 0.672 to 0.795 across validation cohorts. Kaplan–Meier analysis demonstrated significant survival separation between high- and low-risk groups. In addition, Al Mopti et al. (66) introduced a radiomics model based on perirenal fat (PRF) texture features. The combined clinical-radiomics model achieved a C-index of 0.784, outperforming the clinical model alone (C-index 0.653). Time-dependent AUC at 60 months reached 0.8403, indicating favorable long-term predictive performance. Collectively, these studies demonstrate that radiomics and deep learning—particularly when integrating imaging and clinical variables—can enhance prognostic stratification in UTUC. However, heterogeneity in follow-up duration, event rates, and reporting standards underscores the need for standardized methodological frameworks and prospective validation.

An overview of recent studies applying radiomics and machine learning for UTUC prognostic prediction is presented in **Table 1**.

**Table 1:** Overview of studies on radiomics and machine learning models for prognostic prediction in upper tract urothelial carcinoma (UTUC).

Study (year)	N	Study design	Clinical task	Imaging modality	Segmentation	Model type	External validation	Performance
Zheng et al. (55)	140	Retrospective study	Predicting preoperative pathological grade	CTU	Manual segmentation	Random Forest	Yes	AUC (Training Set): 0.914, AUC (Validation Set) 0.903
Nai et al. (56)	215	Retrospective study	Predicting preoperative pathological grade	MRI	Manual segmentation	Gradient Boosting Classifier	No	AUC(Training Set): 1.000 Test AUC(Test Set): 0.786
Al Mopti et al. (57)	103	Retrospective study	Predicting preoperative pathological grade	CT	Semi-automated segmentation	MLPClassifier, CatBoost, Random Forest, etc.	No	AUC(Best tumour grade model:0.961 AUC)Best stage model):0.852
Alqahtani et al., 2024	106	Retrospective study	Predicting tumour grade and stage	CTU	Manual segmentation	Logistic Regression, SVC, MLP, Random Forest, etc.	No	AUC(Tumour Grading):0.94 AUC(Tumour Staging):0.86
Marcon et al. (60)	236	Retrospective study	Differentiation of UTUC from RCC	CT	Manual segmentation	LASSO	Yes	AUC(Training Cohort):0.93 AUC(Test Cohort):0.87
Zhai et al., 2024	80	Retrospective study	Differentiation of UTUC from RCC	CT	Manual segmentation	Random Forest (RF) and combined clinical-radiomics model	Yes	AUC (Training Cohort): 0.99 AUC (Testing Cohort): 0.90
Zhang et al. (62)	163	Retrospective study	Predicting muscle invasion	CTU	Manual segmentation	Logistic Regression (LR) model	Yes	AUC (Training Cohort): 0.859 (95% CI, 0.782–0.917)AUC (Validation Cohort): 0.821
Alqahtani et al., 2024	106	Retrospective study	Predicting survival and recurrence	CTU	Manual segmentation	Cox proportional hazards model	No	C-index (Survival Prediction): 0.731 C-index (Recurrence Prediction): 0.840
Sun et al., 2025	133	Retrospective study	Prognostic prediction	Multi-phase contrast-enhanced CT	Automatic feature extraction	Multi-modal Image-Clinical Combination	Yes	AUC (Training Set): 0.918 AUC (Testing Set): 0.895
Peng et al. (65)	805	Multicenter retrospective cohort study	Prognostic stratification and biomarker exploration	Whole-slide images	UCSegNet tile classifier	Deep learning models	Yes	AUC (UCSegNet): 0.9916–0.9948
Al Mopti et al. (66)	103	Retrospective study	Survival prediction	CTU	Semi-automated segmentation	Clinical model, Radiomics model, Combined model	No	AUC (60 Months): Combined model: 0.8403

The table includes details on study design, clinical tasks, imaging modalities, segmentation methods, model types, external validation, and performance metrics (AUC, C-index) for each study. These studies highlight the use of radiomics features from CTU, MRI, and whole-slide images, alongside clinical data, for predicting various outcomes in UTUC, such as tumor grade, stage, muscle invasion, survival, and recurrence.

## 4 Discussion

Overall, radiomics and deep learning technologies are progressively reshaping the imaging evaluation paradigm of upper tract urothelial carcinoma (UTUC). Across pathological grade prediction, differentiation from renal cell carcinoma, assessment of muscle invasion, and survival stratification, imaging-based artificial intelligence models consistently demonstrate the ability to capture high-dimensional tumor heterogeneity beyond conventional visual interpretation. In several studies, model performance equals or exceeds traditional imaging assessment, and integration of clinical variables, multiphase imaging data, and peritumoral microenvironmental features further enhances discrimination. These findings suggest that imaging phenotypes may reflect not only intrinsic tumor characteristics but also aspects of the tumor microenvironment and potentially underlying molecular biology. As a noninvasive and quantitative framework, artificial intelligence–driven imaging offers promising tools for preoperative risk stratification and individualized treatment planning (67).

Nevertheless, the maturity of current evidence must be interpreted cautiously. The majority of published studies are retrospective and single-center, with limited sample sizes and high-dimensional feature spaces, creating substantial risk of overfitting. In several reports that included independent validation cohorts, performance declined compared with training results, underscoring concerns regarding model robustness and generalizability. Moreover, reliance on ureteroscopic biopsy as the reference standard for tumor grading—despite its known risk of underestimation—may introduce label misclassification. Imaging heterogeneity, subjective manual segmentation, and lack of standardized feature extraction pipelines further compromise reproducibility (68, 69). Many studies do not explicitly address potential data leakage or class imbalance, both of which may distort performance estimates (70, 71).

These issues highlight the critical role of standardization in the application of radiomics and deep learning, particularly in ensuring the consistency and reproducibility of research results. Variations in imaging protocols (such as scanner type, contrast phase, etc.) can lead to inconsistencies in feature extraction, thereby affecting the reliability of the models. Therefore, future studies need to adopt standardized imaging protocols and provide detailed reporting of relevant parameters. At the same time, the variability observed between and within observers during segmentation poses a challenge to model stability. To address this, it is recommended to use intra-class correlation coefficient (ICC) to assess the reproducibility of segmentation and to encourage the use of automated or semi-automated methods to reduce observer bias. Furthermore, feature robustness is central to model reliability, and studies should use ICC to validate the stability of features across different datasets. Finally, adhering to IBSI guidelines for feature extraction will help improve the transparency and comparability of research.

Another critical issue is whether artificial intelligence provides meaningful incremental value beyond experienced radiologists. Although some investigations report superior AUC values compared with visual assessment, prospective head-to-head comparisons remain scarce. Demonstrating additive clinical benefit—rather than statistical improvement alone—is essential before integration into routine workflows can be justified (72, 73). Decision Curve Analysis (DCA), which evaluates clinical utility by considering the clinical consequences of false positives and false negatives, could be used in future studies to assess whether the models provide net clinical benefits across different decision thresholds. This would offer a more meaningful measure of their potential impact in real-world clinical settings.

From a translational perspective, several priorities should be emphasized. First, establishment of multicenter, standardized imaging datasets is necessary to improve robustness and external validity. Second, adherence to methodological quality frameworks such as RQS, TRIPOD-AI, and CLAIM should become routine practice. Third, enhancing interpretability through explainable artificial intelligence techniques may increase clinician trust and facilitate shared decision-making. Fourth, integration of radiomics with molecular pathology and genomic profiling may enable biologically informed multimodal prediction models (74, 75). Finally, prospective real-world studies evaluating the impact of artificial intelligence on treatment decisions, clinical outcomes, cost-effectiveness, and workflow efficiency are urgently needed (76). Randomized clinical trials (RCTs) are also critical to assess whether AI-assisted decision-making leads to improved patient outcomes, including survival and kidney function preservation, as well as cost-effectiveness. Only through rigorous RCTs can we determine whether these models provide tangible benefits for patients.

Regulatory and ethical considerations must also be addressed. Clinical deployment of artificial intelligence tools requires compliance with data protection standards, regulatory approval processes, and clear delineation of medical responsibility. Potential algorithmic bias related to demographic or geographic variation—particularly relevant in UTUC, which demonstrates marked regional incidence differences—must be carefully evaluated to ensure equitable application.

In summary, radiomics and deep learning provide a promising technological foundation for precision management of UTUC. However, most existing models remain at the proof-of-concept stage. Only through methodological standardization, rigorous external validation, prospective evaluation, and interdisciplinary collaboration can artificial intelligence transition from exploratory research to reliable clinical decision-support systems (72).

## 5 Conclusion

Radiomics and deep learning show considerable promise for improving the noninvasive evaluation of upper tract urothelial carcinoma (UTUC), particularly in tumor grading, differential diagnosis, assessment of muscle invasion, and prognostic stratification. By capturing quantitative imaging features beyond conventional visual interpretation, these approaches may support more precise preoperative risk assessment and individualized treatment planning. However, current evidence remains limited by retrospective design, small single-center cohorts, and insufficient external validation. Before routine clinical implementation can be justified, future studies must focus on methodological standardization, prospective multicenter validation, interpretability, and demonstration of clear clinical benefit.
